# The Role of the Immune Response in the Pathogenesis of Thyroid Eye Disease: A Reassessment

**DOI:** 10.1371/journal.pone.0137654

**Published:** 2015-09-15

**Authors:** James T. Rosenbaum, Dongseok Choi, Amanda Wong, David J. Wilson, Hans E. Grossniklaus, Christina A. Harrington, Roger A. Dailey, John D. Ng, Eric A. Steele, Craig N. Czyz, Jill A. Foster, David Tse, Chris Alabiad, Sander Dubovy, Prashant K. Parekh, Gerald J. Harris, Michael Kazim, Payal J. Patel, Valerie A. White, Peter J. Dolman, Deepak P. Edward, Hind M. Alkatan, Hailah al Hussain, Dinesh Selva, R. Patrick Yeatts, Bobby S. Korn, Don O. Kikkawa, Patrick Stauffer, Stephen R. Planck

**Affiliations:** 1 Casey Eye Institute, Oregon Health & Science University, Portland, Oregon, United States of America; 2 Department of Medicine, Oregon Health & Science University, Portland, Oregon, United States of America; 3 Devers Eye Institute, Legacy Health Systems, Portland, Oregon, United States of America; 4 Department of Public Health and Preventive Medicine, Oregon Health & Science University, Portland, Oregon, United States of America; 5 Department of Ophthalmology, Emory University, Atlanta, Georgia, United States of America; 6 Integrated Genomics Laboratory, Oregon Health & Science University, Portland, Oregon, United States of America; 7 Division of Ophthalmology, Ohio University, Athens, Ohio, United States of America; 8 Department of Ophthalmology, The Ohio State University, Columbus, Ohio, United States of America; 9 Department of Ophthalmology, University of Miami, Miami, Florida, United States of America; 10 Department of Ophthalmology, Medical College of Wisconsin, Milwaukee, Wisconsin, United States of America; 11 Department of Ophthalmology, Columbia University, New York, New York, United States of America; 12 Department of Ophthalmology and Visual Sciences, University of British Columbia, Vancouver, BC, Canada; 13 Research Department, King Khaled Eye Specialist Hospital, Riyadh, Saudi Arabia; 14 Ophthalmology Network, Royal Adelaide Hospital, Adelaide, SA 5000, Australia; 15 Department of Ophthalmology, Wake Forrest University, Winston-Salem, North Carolina, United States of America; 16 Department of Ophthalmology, University of California, San Diego, California, United States of America; Baylor College of Medicine, UNITED STATES

## Abstract

**Background:**

Although thyroid eye disease is a common complication of Graves’ disease, the pathogenesis of the orbital disease is poorly understood. Most authorities implicate the immune response as an important causal factor. We sought to clarify pathogenesis by using gene expression microarray.

**Methods:**

An international consortium of ocular pathologists and orbital surgeons contributed formalin fixed orbital biopsies. RNA was extracted from orbital tissue from 20 healthy controls, 25 patients with thyroid eye disease (TED), 25 patients with nonspecific orbital inflammation (NSOI), 7 patients with sarcoidosis and 6 patients with granulomatosis with polyangiitis (GPA). Tissue was divided into a discovery set and a validation set. Gene expression was quantified using Affymetrix U133 Plus 2.0 microarrays which include 54,000 probe sets.

**Results:**

Principal component analysis showed that gene expression from tissue from patients with TED more closely resembled gene expression from healthy control tissue in comparison to gene expression characteristic of sarcoidosis, NSOI, or granulomatosis with polyangiitis. Unsupervised cluster dendrograms further indicated the similarity between TED and healthy controls. Heat maps based on gene expression for cytokines, chemokines, or their receptors showed that these inflammatory markers were associated with NSOI, sarcoidosis, or GPA much more frequently than with TED.

**Conclusion:**

This is the first study to compare gene expression in TED to gene expression associated with other causes of exophthalmos. The juxtaposition shows that inflammatory markers are far less characteristic of TED relative to other orbital inflammatory diseases.

## Introduction

The rationale to classify Graves’ disease as an autoimmune disease is irrefutable. Graves’ disease is characterized by an autoantibody to the thyroid stimulating hormone receptor. The biological activity of this autoantibody results in hyperthyroidism.

It is also widely believed that thyroid eye disease (TED) is autoimmune. First, it frequently coexists with Graves’ disease, so there is “guilt by association”. Many patients with Graves’, however, do not develop TED, nor is it clear that the autoantibody responsible for hyperthyroidism plays a pathogenic role in orbital disease. Thyroid eye disease is associated with polymorphisms in HLA molecules [1] and polymorphisms in receptors that modulate the immune response [2]. An increase in cytokine mRNA expression has been noted in orbital tissue from patients with TED [3]. Cells like fibroblasts or myocytes cultured from the orbit affected by TED display an increase in cytokine synthesis [4, 5]. Elevated levels of circulating cytokines have been detected in patients with TED [6, 7]. Patients with TED have an increased risk for gastrointestinal autoimmunity [8]. Furthermore, many of the strategies aimed at the treatment of TED are based on immunosuppression [9, 10].

In order to clarify the pathogenesis of TED, we have organized a consortium of international centers. We have employed gene expression microarray to analyze the detection of transcripts in orbital tissue from patients with thyroid eye disease. While this type of analysis has been reported previously [11–15], ours is the first report to compare gene expression among different forms of orbital inflammation. By juxtaposing gene expression in TED relative to other causes of orbital inflammation, we find a surprisingly limited immune-response signature relative to several other causes of orbital inflammation.

## Materials and Methods

### Centers and IRB approval

This study was approved by the Institutional Review Board (IRB) at Oregon Health & Science University and at each of the other contributing centers: Columbia University IRB, University of California San Diego IRB, Wake Forest University IRB, Medical College of Wisconsin IRB, Mount Carmel (Ohio) IRB, University of Miami IRB, University of British Columbia Clinical Research Ethics Board, Royal Adelaide Hospital Research Ethics Committee, King Khaled Eye Specialist Hospital Human Ethics Committee/Institutional Review Board. Tissue acquisition was in compliance with the requirements of the local review board including acquisition of written informed consent from the donor where required. All data were anonymized and de-identified prior to analysis. The research adhered to the tenets of the Declaration of Helsinki.

Formalin-fixed, paraffin-embedded (FFPE) samples and relevant demographic and clinical data were obtained. The diagnoses of sarcoidosis, granulomatosis with polyangiitis (GPA) (previously known as Wegener’s granulomatosis), TED, nonspecific orbital inflammation (NSOI) (previously also known as orbital pseudotumor or idiopathic orbital inflammation), and normal were based on the clinical and histopathological information obtained and submitted by orbital disease specialists and ocular pathologists from their respective institutions. All biopsies were further reviewed by two of the authors (DJW and HEG) as noted below.

This is a multicenter, international study on previously biopsied orbital tissue. Biopsies of orbital adipose tissue from a total of 83 subjects were studied (20 controls with no known orbital disease, 6 with GPA, 7 with sarcoidosis, 25 with TED, and 25 with NSOI). The ages are summarized in Table 1. The gender distribution was roughly comparable among the 5 groups. Seventy-six per cent of the TED patients were female, compared to 64% of those with NSOI, 83% of those with sarcoidosis, 67% of those with GPA, and 70% of the healthy controls. Of the 25 patients with TED, 15 were biopsied prior to starting therapy, four had received prior corticosteroid therapy, and one had received local radiation. Prior therapy information was not provided on 5 patients. Nineteen of the 25 patients were known to be hyperthyroid, one was hypothyroid, and 3 were euthyroid. Thyroid status was not provided on two subjects. Twenty of the patients had orbital surgery for symptomatic relief. The reason for surgery was not stated in 5 subjects. Thyroid antibody status was reported in 5 patients and was positive in 3 of these. The control tissue was obtained during surgery such as blepharoplasty and enucleation on eyes with non-inflamed orbits.

**Table 1:** Ages for each experimental group.

	Set 1		Set 2	
Diagnosis	N	Mean age at biopsy	N	Mean age at biopsy
NSOI	14	44.0 ± 21.8	11	58.5 ± 24.1
Sarcoid	1	60.6	7*	48.8 ± 14.7
TED	14	54.0 ± 14.7	11	48.6 ± 13.0
GPA	4	43.4 ± 14.3	4**	40.0 ± 13.9
Normal	14	61.0 ± 15.6	6	69.7 ± 9.8

*one repeated from set 1;

**two repeated from set 1

### Pathology Review

Hematoxylin and eosin stained slides of all samples were evaluated in a masked fashion by two ocular pathologists (D.J.W. and H.E.G.) for histopathological characteristics. The diagnosis from the pathologists was compared to the diagnosis from the institution where tissue had been obtained. In some cases, additional stains were requested or additional clinical information was reviewed. A few cases with an ambiguous diagnosis were excluded. In all cases included in this study, a consensus diagnosis was obtained by Drs. Wilson and Rosenbaum, and the contributing center’s diagnosis was accepted.

### Tissue Preparation and Gene Expression Profiling

RNA extraction and microarray assays were performed in the OHSU Gene Profiling Shared Resource as previously reported [16]. We used Human U133 Plus 2.0 arrays that contain over 54,000 probe sets for 47,000 human transcripts and variants.

### Statistical Analysis

Affymetrix CEL files were normalized by the Robust Multiarray Analysis [17]. Testing for potential differences in gene expression across disease groups was done by fitting linear models after controlling for sex and age at biopsy. Mean differences were considered statistically significant when there was at least 1.5-fold change with a false discovery rate adjusted p-value less than 0.05. The ‘affy’, ‘caret’ ‘MASS’, ‘limma’ and ‘randomForest’ packages in the R project (http://www.r-project.org) were used for statistical computations.

Principal component analysis (PCA) was used for two dimensional or three-dimensional visualization of the dissimilarity in gene expression profiles across the disease groups [18]. A dendrogram that could be aligned with heat maps was created by unsupervised, hierarchical cluster analysis. The data used for the PCA plots and dendrogram were restricted to those probe sets that had significantly different signals for TED, sarcoidosis, GPA, or NSOI groups compared to normal controls. The GSEA program [19, 20] provided a means to identify expression differences in predetermined sets of genes without reliance on the FDR p-values and fold-differences used to select the probe sets employed in the creation of the dendrograms. The data used for the generation of the heat maps were restricted to 40,450 probe sets with adequate annotation and custom gene lists for immunoglobulins, cytokines, cytokine receptors, chemokines, and chemokine receptors.

## Results

Table 1 shows the mean ages for control groups and for subjects with TED in this study. Because studies based on multiple statistical comparisons are fraught with “significant” findings that merely represent chance occurrences from multiple statistical tests, we divided our subjects into a discovery and a validation set. The validation set determines if the differences found in the discovery set are reproducible.

**Fig 1:**
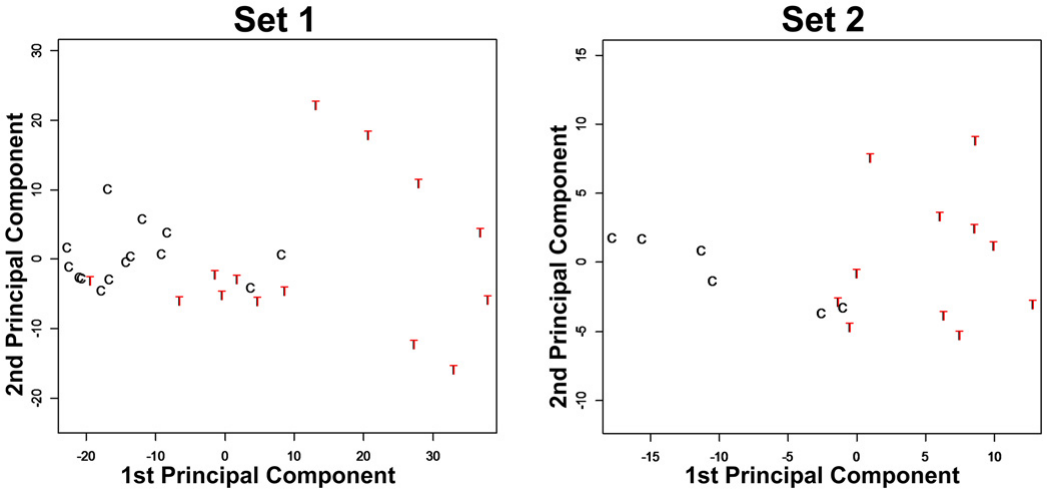
Principal component analysis based on significantly up-regulated and significantly down-regulated probe sets in TED orbital adipose (T) against uninflamed controls (C). The discovery set (Set 1) had 36 significantly up-regulated and 254 significantly down-regulated probe sets. The validation set (Set 2) had 66 significantly up-regulated and 604 significantly down-regulated probe sets. At least 1.5-fold change with FDR adjusted p-value < 0.05 is considered statistically significant.

Fig 1 shows principal component analysis (PCA) plots that compare the difference in gene expression from TED patients and from healthy controls. In a PCA, the distance between two points reflects their relative similarity or dissimilarity. For the discovery set (set 1), the separation between the two groups indicates that there are clear differences between TED and controls. For the validation set (set 2), there is again a grouping of TED distinct from the controls. However, only a minority of the probe sets that distinguished TED from controls in the discovery set were reproduced in the validation set. In fact, no probe sets indicated transcripts with a significantly higher signal (>1.5-fold increase with a false discovery rate adjusted p value of <0.05) in both the discovery set and the validation set in a comparison between TED and the healthy controls. Table 2 lists the 29 probe sets (~23 genes) that had significantly lower signals in tissue from patients with thyroid eye disease compared to tissue from non-diseased controls. Thus, TED is distinct from controls, but the distinction is not strong.

**Table 2:** Probe sets with a significantly lower signal in orbital tissue from TED subjects compared to uninflamed controls.

			Set 1		Set 2	
Probe Set	Gene Symbol	Gene Title	Fold Difference	FDR p-value	Fold Difference	FDR p-value
203305_at	F13A1	coagulation factor XIII, A1 polypeptide	-3.87	0.012	-3.10	0.019
213800_at	CFH	complement factor H	-3.52	0.022	-3.05	0.002
207277_at	CD209	CD209 molecule	-4.25	0.006	-2.24	0.027
203407_at	PPL	periplakin	-2.69	0.025	-3.46	0.009
209348_s_at	MAF	v-maf musculoaponeurotic fibrosarcoma oncogene homolog (avian)	-3.56	0.046	-2.54	0.040
201212_at	LGMN	legumain	-3.62	0.007	-2.38	0.005
215039_at	LOC339524	uncharacterized LOC339524	-3.75	0.011	-2.16	0.024
237351_at	LOC100652994	uncharacterized LOC100652994	-3.73	0.000	-2.03	0.028
215870_s_at	PLA2G5	phospholipase A2, group V	-3.30	0.002	-2.15	0.019
1554485_s_at	TMEM37	transmembrane protein 37	-3.46	0.001	-1.88	0.023
240873_x_at	DAB2	Dab, mitogen-responsive phosphoprotein, homolog 2 (Drosophila)	-2.79	0.029	-2.40	0.005
1555729_a_at	CD209	CD209 molecule	-2.51	0.009	-2.27	0.028
203414_at	MMD	monocyte to macrophage differentiation-associated	-2.64	0.036	-2.07	0.045
213001_at	ANGPTL2	angiopoietin-like 2	-2.60	0.013	-2.02	0.041
232712_at	---	---	-2.70	0.044	-1.82	0.031
209543_s_at	CD34	CD34 molecule	-2.31	0.043	-2.11	0.049
228347_at	SIX1	SIX homeobox 1	-2.37	0.036	-2.00	0.034
206856_at	LILRB5	leukocyte immunoglobulin-like receptor, subfamily B (with TM and ITIM domains), member 5	-2.74	0.008	-1.61	0.030
231846_at	FOXRED2	FAD-dependent oxidoreductase domain containing 2	-2.23	0.039	-2.10	0.012
226841_at	MPEG1	macrophage expressed 1	-2.26	0.031	-2.05	0.020
206178_at	PLA2G5	phospholipase A2, group V	-2.27	0.003	-1.82	0.024
206726_at	HPGDS	hematopoietic prostaglandin D synthase	-2.17	0.006	-1.89	0.008
244111_at	KRT222	keratin 222	-2.39	0.023	-1.65	0.043
238151_at	---	---	-2.21	0.003	-1.74	0.029
204829_s_at	FOLR2	folate receptor 2 (fetal)	-2.25	0.003	-1.57	0.040
222876_s_at	ADAP2	ArfGAP with dual PH domains 2	-2.10	0.012	-1.69	0.035
225228_at	DRAM2	DNA-damage regulated autophagy modulator 2	-1.88	0.048	-1.60	0.031
218032_at	SNN	stannin	-1.68	0.020	-1.51	0.024
218633_x_at	ABHD10	abhydrolase domain containing 10	-1.53	0.031	-1.50	0.048

**Fig 2:**
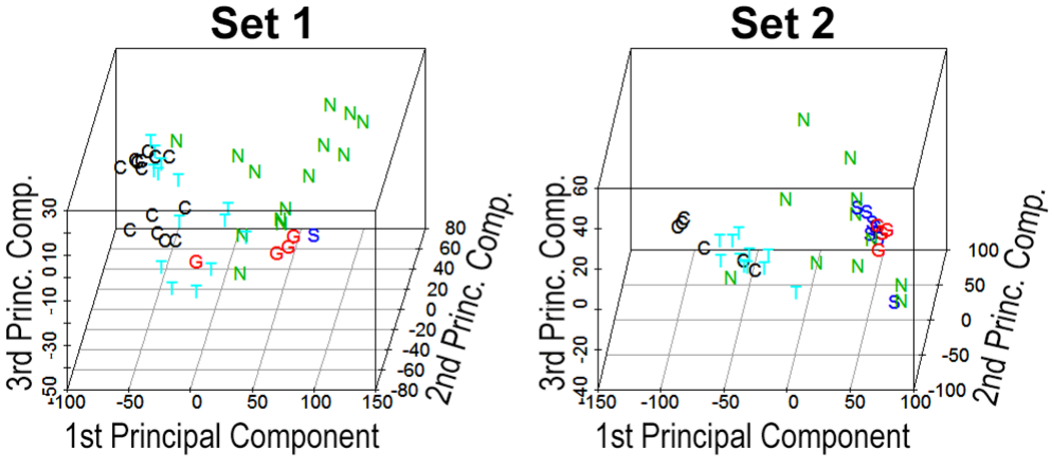
Principal component analysis reveals that gene expression in TED orbital adipose is more similar to uninflamed controls than to tissues from subjects with sarcoidosis, GPA, or NSOI. C = control. T = TED. N = NSOI. S = Sarcoidosis. G = GPA. Set 1 is based on the discovery set and Set2 is based on the validation set.

The PCA plots in Fig 2 compare gene expression in orbital tissue from patients with TED to orbital tissue from patients with sarcoidosis, NSOI, or GPA. Also included are subjects with no known history of orbital disease. The grouping clearly shows that TED is much more similar to control tissue compared to subjects with sarcoidosis, most subjects with NSOI, or patients with GPA.

**Fig 3:**
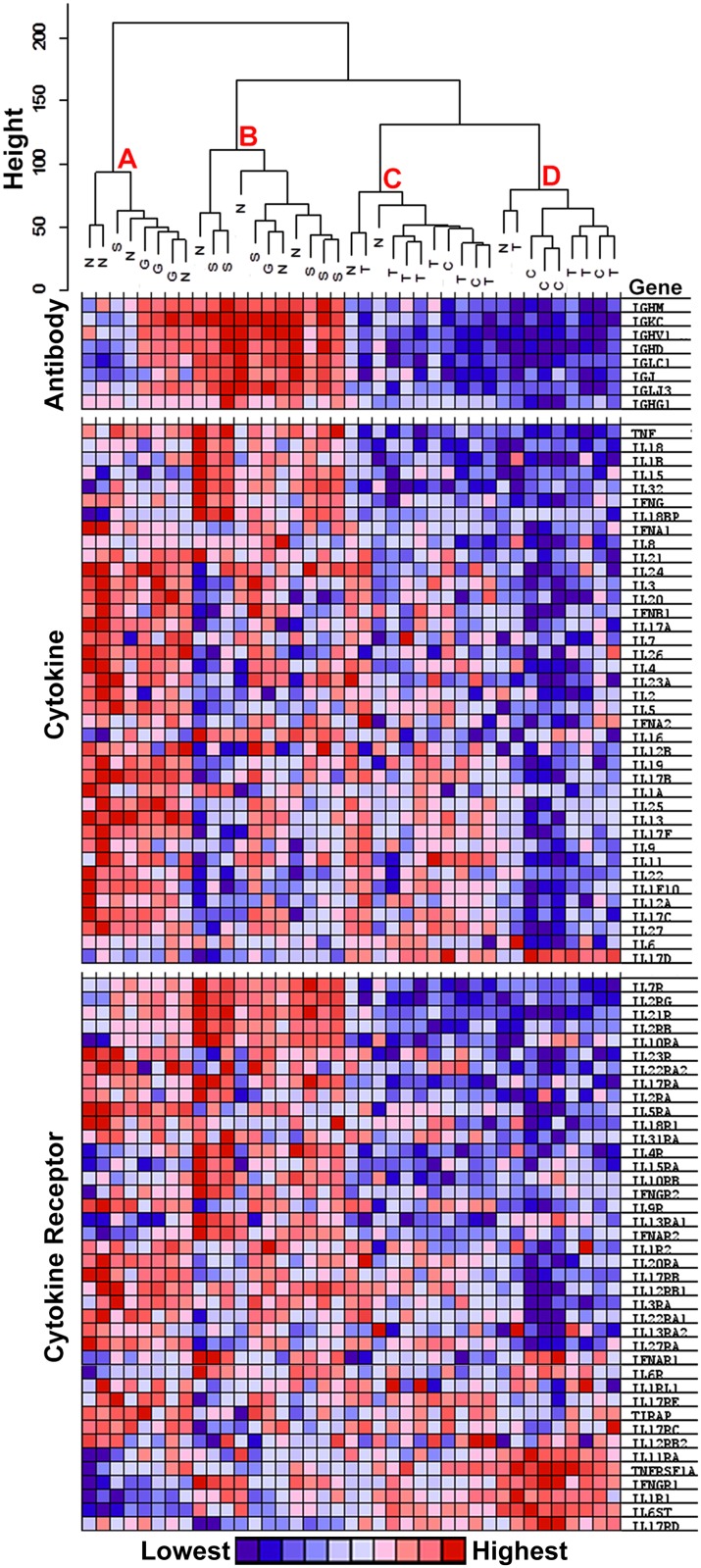
A dendrogram produced by hierarchical cluster analysis and an independently created heat map of signals for immunoglobulin, cytokine, and cytokine receptor transcripts compares the expression profiles of subjects within each disease group in array dataset 2. The heat maps were created with the GSEA program comparing probe set signal levels for subjects in branches A and B versus branches C and D. Genes with the highest signals in branches A and B are at the top of each section. The end branch labels are N—NSOI, S—sarcoidosis, G—GPA, T—TED, C—control.

**Fig 4:**
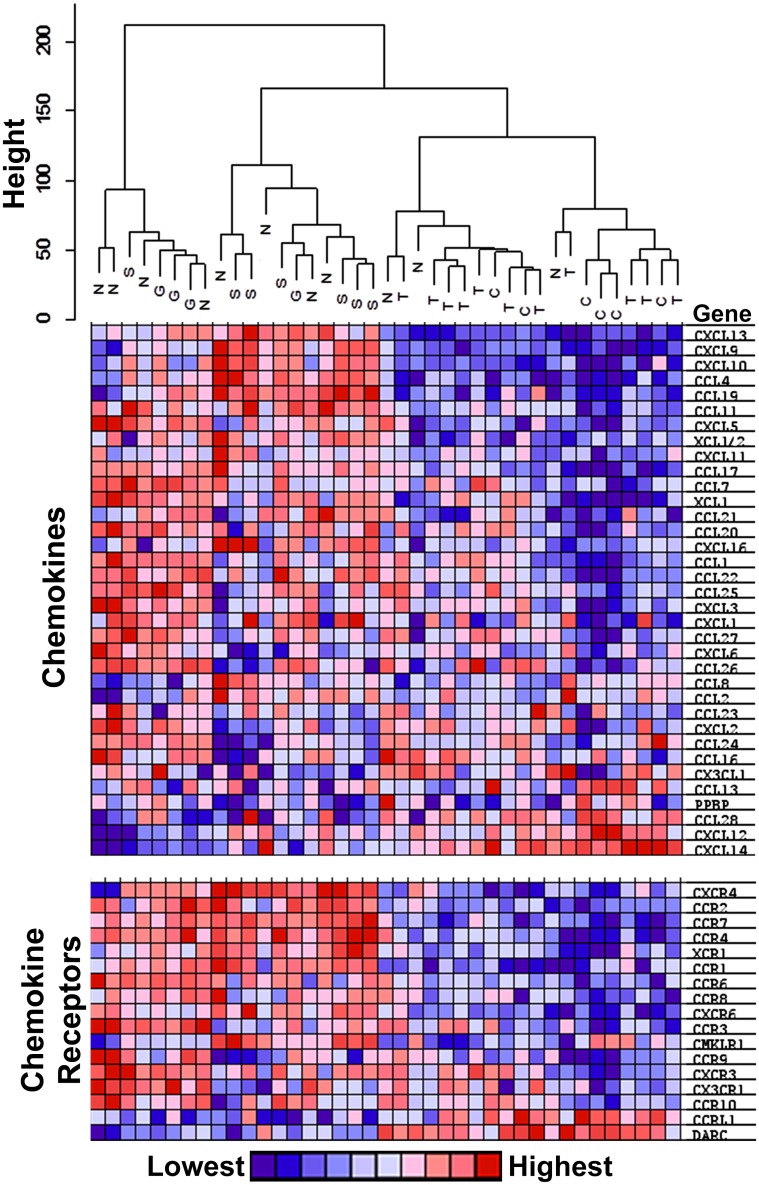
Heat map showing relative chemokine and chemokine receptor expression aligned with the same dendrogram shown in Fig 3.

Another way to represent the correspondence of complex data sets is to display the results as an unsupervised cluster dendrogram in which the height of the branches reflects the relative difference between the branches. The dendrograms at the top of Figs 3 and 4 are based on the validation set and indicate that subjects with TED display gene expression patterns that cluster with patterns for uninflamed controls in the branches labeled C and D. In contrast, the patterns for subjects with sarcoidosis or GPA are found in the A and B branches. The NSOI subject patterns are distributed more widely, but only a minority is in the C and D branches. Dendrograms for a similar analysis of the discovery set data are shown in S1 and S2 Figs. The dendrograms indicate that the TED expression pattern is more closely aligned to healthy tissue than to diseased tissue from patients with sarcoidosis, GPA, or NSOI.

The heat maps below the dendrograms indicate the relative expression of some immunologically important transcripts: immunoglobulins, cytokines, and cytokine receptors in Fig 3 and chemokine and chemokine receptors in Fig 4. The heat map columns are aligned with the corresponding samples in the dendrogram. Heat maps for a similar analysis of the discovery set data are shown in S1 and S2 Figs. These figures indicate clearly that diseases such as GPA, sarcoidosis, and NSOI are much more associated with increased expression of these transcripts in comparison to TED.

We considered a variety of potential confounders for this study including whether the biopsy was obtained prior to therapy or subsequent to therapy and whether the patient was on corticosteroids at the time of biopsy. Surprisingly neither of these variables appears to be contributing to the results.

## Discussion

A recent review from respected authorities on TED concluded: “lymphocytes and cytokines are intimately involved in the initiation, amplification, and maintenance of the autoimmune process in TED.” [21] TED is widely considered to be an immune-mediated disease. Observations that support this include its improvement with prednisone or B cell depleting therapy [22], its co-existence with Graves’ disease which is driven by autoantibodies, and increased expression of cytokines in the affected orbital tissue [14, 23, 24]. However, a randomized controlled trial using B cell depletion failed to demonstrate benefit for TED [25]. The therapeutic effect of prednisone is nonspecific since corticosteroids regulate many genes in addition to those involved in immune responses [26]. In parallel with our investigation of TED, we have collected tissue from patients with additional forms of orbital inflammation including sarcoidosis, GPA, and NSOI. Juxtaposing gene expression from TED with gene expression from 3 other causes of exophthalmos shows that inflammatory markers are much less commonly associated with TED relative to the other two diagnoses.

We recently reported on the immunohistochemical detection of IgG4 in biopsies from patients with either orbital or lacrimal disease [27]. IgG4 is widely accepted as a marker of immune activation [28]. We detected IgG4 in 30% or more of subjects with GPA, sarcoidosis, or NSOI. However, substantial IgG4 staining was consistently absent in tissue from subjects with TED or healthy controls. These observations further support the contention that only a low level of inflammation generally accompanies thyroid eye disease. Our studies do not mean that the immune response has no role in the pathogenesis of TED. However, its role is relatively slight, suggesting that metabolic or other pathways might be better therapeutic targets to treat TED.

Several previous groups have attempted to clarify the pathogenesis of TED using broad amplification techniques similar to what we describe [11–15]. Our approach has advantages over prior studies. It is the first to be able to make comparisons to tissue representing other forms of orbital inflammation. It is the first to include both a discovery and a validation set. We have previously validated our microarray data using quantitative PCR and have shown a correlation of greater than 0.7 between the PCR and microarray values.

We also acknowledge limitations. While the diagnosis of TED is rarely controversial, we have relied on the clinical expertise from multiple centers such that we cannot exclude some diagnostic heterogeneity. Our gene expression data, however, support that we have studied a fairly uniform disease entity. As stated in the methods, about 60% of the patients with TED in this study were biopsied prior to starting therapy. Most of the biopsies were performed one or more years after disease onset. It is possible, therefore, that many of the biopsies were obtained after an early phase of TED that may have a more active, immune-mediated component. It is also possible that biopsy of extra-ocular muscle would have revealed an inflammatory signature that we could not detect in adipose tissue. Relief of symptoms was the motivation for most of the TED biopsies, whereas biopsies of the other diseases were primarily for diagnostic purposes. This difference might lead to the other diseases being sampled during an earlier, stage of disease, although in prior published analysis of gene expression in orbital inflammatory disease we could not demonstrate that disease duration affected levels of transcripts [29]. Our sample numbers were too small to test for differences due to demographic variables, such as age and gender, so to compare the disease groups we used linear models after controlling for these variables. We are in the process of collecting additional biopsies to assess the role of disease duration, therapy, and site of biopsy more thoroughly. We also acknowledge that artifacts can result from multiple statistical comparisons.

Some patients with thyroid eye disease are euthyroid. Others develop orbital disease well in advance of clinical thyroid disease. We believe that this approach to gene expression profiling will complement any information that can be gleaned by light microscopy such that ultimately orbital disease will be diagnosed in part by gene expression profiling.

An analysis of the molecular pattern of gene expression in TED also has the potential to clarify pathogenesis and thus to suggest new forms of therapy. Furthermore, some patients with TED have disease which is especially protracted. We predict that gene expression will help to identify subsets of patients such as those whose disease is especially aggressive or persistent or those who are especially likely to benefit from specific forms of therapy. Our observations support an approach to therapy that is not based solely on immunosuppression.

## Supporting Information

S1 Fig A dendrogram produced by hierarchical cluster analysis and an independently created heat map of signals for cytokine and cytokine receptor transcripts compares the expression profiles of subjects within each disease group in array dataset 1. The end branch labels are N—NSOI, S—sarcoidosis, G—GPA, T—TED, C—control.
(PDF)

S2 Fig A dendrogram produced by hierarchical cluster analysis and an independently created heat map of signals for chemokine and chemokine receptor transcripts compares the expression profiles of subjects within each disease group in array dataset 1. The end branch labels are N—NSOI, S—sarcoidosis, G—GPA, T—TED, C—control.
(PDF)
