# Supplementary material for: The Role of the Immune Response in the Pathogenesis of Thyroid Eye Disease: A Reassessment
Source: PLoS One. 2015 Sep 15;10(9):e0137654. doi: 10.1371/journal.pone.0137654 (PMC4570801; doi:10.1371/journal.pone.0137654)

Figure S2. A dendrogram produced by hierarchical cluster analysis and an independently created heat map of signals chemokine and chemokine receptor transcripts compares the expression profiles of subjects within each disease group in array dataset 1. The end branch labels are N – NSOI, S – sarcoidosis, G – GPA, T – TED, C – control.

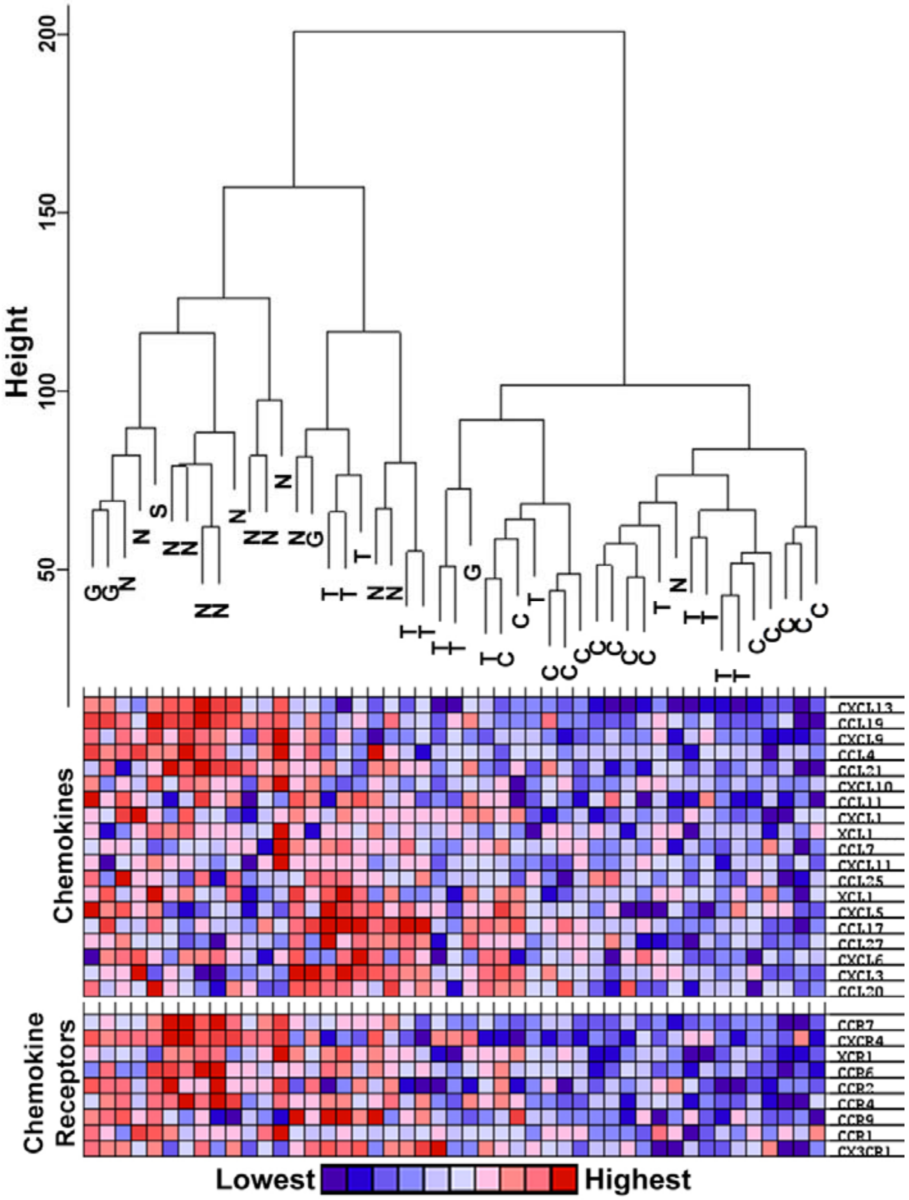

Supplement: S2 Fig — The end branch labels are N—NSOI, S—sarcoidosis, G—GPA, T—TED, C—control. (PDF) [file pone.0137654.s002.pdf]
